# Extirpation of More Than Half the Lower Jaw, for a Cancerous Degeneration

**Published:** 1852-01

**Authors:** Frank H. Hamilton

**Affiliations:** Professor of Surgery in the University of Buffalo, and One of the Surgeons to the Buffalo Hospital of the Sisters of Charity


ART. II.—*Extirpation of more than half the Lower Jaw, for a Cancerous Degeneration.* By Frank H. Hamilton, Professor of Surgery in the University of Buffalo, and one of the Surgeons to the Buffalo Hospital of the Sisters of Charity.

Dewitt C. Hooper, aged 49 years, was admitted to this hospital, as a private patient, January 24, 1851.

In 1844, he first discovered a small movable tumor under the right ramus of the lower jaw. One year since a cancer doctor cauterized it, and destroyed the tumor with some of the adjacent structure. The wound thus made healed slowly, and left new indurations, which rapidly extended, and the bone became involved.

The disease, at the time of the operation, implicated the whole right half of the inferior maxilla and much of the neighboring soft parts. His health was good.

*Operation.*—Jan. 25, 1851. Patient under the influence of chloroform. An incision was made from near the glenoid cavity, along the descending and horizontal ramus of the symphysis. This incision was intersected by a second, commencing at the angle of the mouth and terminating at the angle of the jaw. The flaps thus made being lifted, the bone was separated about five lines to the left of the symphysis, with a chain saw, and the condyle was then removed from its socket. The operation was not commenced with the expectation of removing so large a portion of the jaw, but, on exposure, and having tried it with the knife, it was found diseased in its cancelli, from the socket to the symphysis. The soft parts were also largely involved both within and without the mouth. As effectually as possible the whole was cleared away. Three vessels only required the ligature. The wound was closed with sutures.

The wound granulated and continued to heal steadily until the 22d of February. An opening of half an inch in diameter still existed, through which healthy matter was discharged; his strength was such that he sat up a considerable portion of the day; his appetite was good, and a favorable issue seemed probable if not certain.

On the morning of the 22d he arose as usual to take his breakfast, and intending, after breakfast, to leave the hospital, when he was suddenly seized with a sensation of unusual prostration; he was laid upon the bed, and died in a few moments. No medical officer was called, and the body having been removed soon after, no autopsy was made. The cause of his death remains, therefore, unexplained.

